# Comparative Transcriptome Analysis Provides New Insights into the Molecular Regulatory Mechanism of Adventitious Root Formation in Ramie (*Boehmeria nivea* L.)

**DOI:** 10.3390/plants10010160

**Published:** 2021-01-15

**Authors:** Kunmei Chen, Bing Guo, Chunming Yu, Ping Chen, Jikang Chen, Gang Gao, Xiaofei Wang, Aiguo Zhu

**Affiliations:** Institute of Bast Fiber Crops, Chinese Academy of Agricultural Sciences, No. 348, West Xianjiahu Road, Changsha 410205, China; chenkunmei@caas.cn (K.C.); guobing@caas.cn (B.G.); yuchunming@caas.cn (C.Y.); chenping02@caas.cn (P.C.); cjk213@126.com (J.C.); gaogang@caas.cn (G.G.); xiaofei1008@126.com (X.W.)

**Keywords:** ramie (*Boehmeria nivea* L.), adventitious roots, hydroponics, mRNA-Seq, transcriptomic divergence

## Abstract

The occurrence of adventitious roots is necessary for the survival of cuttings. In this study, comparative transcriptome analysis between two ramie (*Boehmeria nivea* L.) varieties with different adventitious root (AR) patterns was performed by mRNA-Seq before rooting (control, CK) and 10 days water-induced adventitious rooting (treatment, T) to reveal the regulatory mechanism of rooting. Characterization of the two ramie cultivars, Zhongzhu No 2 (Z2) and Huazhu No 4 (H4), indicated that Z2 had a high adventitious rooting rate but H4 had a low rooting rate. Twelve cDNA libraries of the two varieties were constructed, and a total of 26,723 genes were expressed. In the non-water culture condition, the number of the distinctive genes in H4 was 2.7 times of that in Z2, while in the water culture condition, the number of the distinctive genes in Z2 was nearly 2 times of that in H4. A total of 4411 and 5195 differentially expressed genes (DEGs) were identified in the comparison of H4CK vs. H4T and Z2CK vs. Z2T, respectively. After the water culture, more DEGs were upregulated in Z2, but more DEGs were downregulated in H4. Gene ontology (GO) functional analysis of the DEGs indicated that the polysaccharide metabolic process, carbohydrate metabolic process, cellular carbohydrate metabolic process, cell wall macromolecule metabolic process, and photosystem GO terms were distinctively significantly enriched in H4. Simultaneously, Kyoto Encyclopedia of Genes and Genomes (KEGG) analysis showed that photosynthesis, photosynthesis antenna proteins, and starch and sucrose metabolism pathways were distinctively significantly enriched in H4. Moreover, KEGG analysis showed that jasmonic acid (JA) could interact with ethylene to regulate the occurrence and number of AR in Z2. This study reveals the transcriptomic divergence of two ramie varieties with high and low adventitious rooting rates, and provides insights into the molecular regulatory mechanism of AR formation in ramie.

## 1. Background

The rooting ability of clones is the key factor to determine the success of the propagation of cuttings, and it is also the main criterion for distinguishing the adaptability and resistance of clones. Adventitious root (AR) is the root of cuttage plants which is an important organ for water and inorganic salt absorption and assimilate storage, and plays an important role in stress resistance [1,2]. The occurrence of AR enlarges the root system of plants and enables plants and cells to regenerate. In most plants, AR originates from flood conditions. When plants suffer from hypoxia or other stress, AR can significantly improve the survival ability of plants [3]. Therefore, in-depth understanding of the mechanism of AR is helpful to improve the survival rate of cuttings and the survival ability of plants under stress.

Ramie (*Boehmeria nivea* L.), which has a history of over 4000 years in China, is currently mainly planted in China, India, and other Southeast Asian and Pacific Rim countries. It can be used as an excellent fiber crop, a forage plant, a soil and water conservation plant, and as an ideal crop for cadmium pollution remediation [4,5]. Ramie is a highly heterozygous crop [6], thus, it is impossible to maintain the fine quality of ramie by seed propagation, but asexual propagation can solve the problem well. Asexual propagation is faster than seed reproduction, and can maintain the genetic stability of cultivars, which make it a popular way of propagation in potato, strawberry, grape, kiwifruit, chestnut, and ramie. With the development and utilization of ramie, the demand of raw ramie materials is increasing. Asexual propagation can rapidly propagate a large number of ramie seedlings to meet the needs of production.

AR formation is of utmost interest since it consists of the development of root tissues from non-root and non-meristematic tissues. The formation and development of AR are regulated by various biological processes, and its molecular regulative mechanism is complex. At present, it has been found that plant hormones play an important role in AR formation. Indole-3-butyric acid can act as a central player in the hormone crosstalk that controls adventitious rooting in many plants such as *Pinus thunbergii*, *Camellia sinensis*, and *Sterculia foetida* [7,8,9,10]. In cucumber, abscisic acid can induce adventitious rooting of its explants under drought stress [11]. In tomato, ethylene plays a positive role in AR formation and a negative role in lateral root formation with modulation of auxin transport as a central point of ethylene–auxin crosstalk [12]. In petunia, ethylene and auxin act as regulators during AR formation of petunia cutting [13]. In hybrid aspen and *Arabidopsis*, gibberellin can inhibit adventitious rooting by affecting auxin transport [14]. In *Morinda citrifolia* and apple, cytokinin can inhibit AR primordia formation and AR growth [15,16]. In *Arabidopsis* and pea, strigolactone suppresses adventitious rooting but promotes it in rice [17,18]. However, in ramie, whether plant hormones affect AR in the cuttings is unknown. The molecular mechanism underlying adventitious rooting in ramie cutting propagation is still elusive.

mRNA-Seq and comparative transcriptome have been popularly used to explore pathways during AR formation and development [19,20,21]. This study reports the transcriptomic analysis of two ramie varieties Zhongzhu No2 (Z2) and Huazhu No4 (H4) with a distinct rooting rate to uncover the molecular mechanisms regulating adventitious rooting in ramie cuttings. We aimed to identify the key pathways involved in this process. This study will be helpful to improve the survival rate of ramie cuttings.

## 2. Methods

### 2.1. Ethics Statement

The ramie varieties used in this study were planted in our scientific research field, which is owned by our institution. Therefore, no specific permissions were required for using these plant materials.

### 2.2. Plant Growth and Sampling

Two ramie cultivars, Zhongzhu No 2 (Z2) and Huazhu No 4 (H4), were used in this study. The ramie plants grew in a greenhouse at 25 ± 1 °C with a 12 h photoperiod, 75% ± 1 RH, under light intensity of 5000 lux. Shoots of 10–15 cm in length were collected and then inserted into conical flasks with tap water, one shoot in one flask, and there were about 4–5 cm of the stem submerged in the water. The tap water was renewed every two days. The number of root primordia was counted every day. Ten and 20 days after water culture, stem segments submerged in water (with root primordium) were collected as treatment samples (T). Meanwhile, stems cut from the mother plants without water treatment were collected as control samples (CK). All samples were immediately frozen in liquid nitrogen and stored at −80 °C. Three biological replicates were set for CK and T samples, and each replicate contained five individual plants.

### 2.3. RNA Isolation, Library Preparation and Sequencing

Control samples and samples treated with tap water for 10 days were used in transcript sequencing. Total RNA was extracted from the samples with TRIzol Reagent (Invitrogen, Carlsbad, CA, USA) and purified with Plant RNA Purification reagent (Invitrogen, Carlsbad, CA, USA) according to the manufacturer’s protocol. The concentration and purity of total RNA was determined using a NanoDrop2000 spectrophotometer (Thermo Fisher Scientific, Waltham, MA, USA), and RNA integrity was examined by an Agilent2100 bioanalyzer (Agilent, Santa Clara, CA, USA).

For transcript sequencing, 12 libraries (Z2CK1, Z2CK2, Z2CK3, Z2T1, Z2T2, Z2T3, H4CK1, H4CK2, H4CK3, H4T1, H4T2, and H4T3) were constructed using 5 μg of total RNA. Briefly, poly (A) mRNA was extracted from the total RNA sample using Oligo (dT) magnetic beads. mRNA was fragmented into 200–500 nt pieces by adding a fragmentation buffer. First-strand cDNA was synthesized using SuperScript II reverse transcriptase (Life Technologies, Inc., Carlsbad, CA, USA) and random hexamer primers. After generation of second-strand cDNA, the double-strand cDNA was end-repaired, and a single ‘A’ base and indexed adapters were ligated to the fragments. The cDNA library was constructed with an Illumina Paired End Sample Prep kit (Illumina, San Diego, CA, USA), quantified by TBS380, (Picogreen, Invitrogen, Carlsbad, CA, USA) and was then sequenced on the Illumina HiSeq^TM^2500 (2 × 150 bp read length) platform. Illumina sequencing was performed at Majorbio Bio-pharm Technology Co. Ltd., Shanghai, China (http://www.majorbio.com/). Illumina sequencing data was deposited in the NCBI GEO database with accession number GSE116063.

### 2.4. Data Filtering and Mapping

Adapter sequences, low quality reads (Q < 20), extreme short sequences, and reads with unknown nucleotides larger than 5% were removed to obtain high-quality reads. The remaining reads were classified as clean reads. The clean reads were mapped to the ramie reference genome (a chromosome-scale, high-quality reference genome of ramie, assembled with an excellent ramie variety Zhongzhu NO 1, data unpublished, review link: https://db.cngb.org/cnsa/project/CNP0001467/reviewlink.) using HISAT2 (v2.1.0, http://www.ccb.jhu.edu/software/hisat).

### 2.5. Identification of Differentially Expressed Genes (DEGs)

Gene expression levels of the mapped reads were represented by the expected number of fragments per kilobase of transcript sequence per millions base pairs sequenced (FPKM), which was calculated by RSEM (v1.2.8, http://deweylab.biostat.wisc.edu/rsem/rsem-calculate-expression.html). DEGs were identified using the DESeq2 R package according to the method described by [22]. The resulting *p*-values were adjusted using Benjamini and Hochberg’s approach for controlling the false discovery rate. Genes were designated as differentially expressed when the adjusted *p*-value was <0.001 and estimated absolute log2 (fold change) ≥ 2. The software VennDiagram (version: 1.6.20) was used to draw the Venn diagram of all the genes and the DEGs.

### 2.6. Gene Ontology (GO) and Pathway Enrichment Analysis of DEGs

GO enrichment analysis of DEGs was performed using the cluster Profiler R package. GO terms with an adjusted *p*-value < 0.05 were considered to be significantly enriched, and were categorized into three types of functional classification, namely cellular component, molecular function, and biological process. Pathway analysis was performed using the Kyoto Encyclopedia of Genes and Genomes (KEGG) web server (https://www.kegg.jp/), and enrichment analysis was conducted by the cluster Profiler R package. Pathways with an adjusted *p*-value < 0.05 were considered to be significantly enriched.

### 2.7. Quantitative PCR (qPCR) Validation of DEGs

Control samples and samples treated with tap water for 10 and 20 days were used in qPCR analysis. Briefly, 0.5 μg total RNA was used to synthesize cDNA with oligo (dT) using a PrimeScript RT perfect real-time reagent kit (Takara, Japan), and then the cDNA was diluted four times. The qPCR analysis was performed using a CFX96 Real-Time System (Bio-Rad) with the special primers showed in Additional file 1: sheet1, according to the method of Chen et al. [23]. Briefly, the reaction systems contained 10 μL 2 × T5 Fast qPCR Mix (SYBR Green I, TSINGKE Biological Technology, Beijing, China), 0.4 μmol/L of each of the forward and reverse primers, 2 μL diluted cDNA, and an appropriate amount of sterile double-distilled water. The 18S rRNA gene was used as the internal control to normalize q-PCR data. Samples without water culture were set as control, and the relative expression of target genes of samples with water culture for different days was calculated using the 2^−ΔΔCt^ method [24].

## 3. Results

### 3.1. Adventitious Root Formation of Two Ramie Cultivars

**Figure 1:**
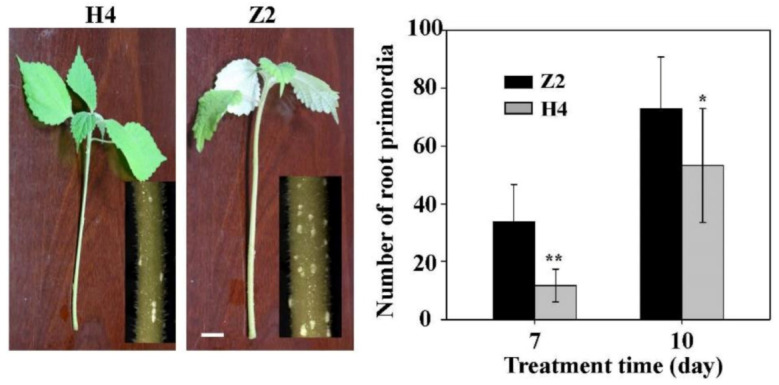
Phenotypic analysis of ramie cuttings cultured with tap-water. H4 and Z2 represent Huazhu No 4 and Zhongzhu No 2, respectively. The left pictures show the ramie cuttings cultured with tap water for 7 days; white bar = 1 cm. The right column chart is the statistical analysis of the number of root primordia of ramie cuttings cultured with tap water. Statistical analysis was conducted using one-way analysis of variance with SPSS Statistics 19.0 (SPSS Inc., Chicago, IL, USA). Comparisons of means were performed using least significant difference test at *p* = 0.05. * and ** denote significant difference from Z2 at *p* < 0.05 and *p* < 0.01, respectively. Data are means ± SD of three replicates. Each replicate included five individual plants.

In order to study the rooting rate of Z2 and H4, root primordia formation in the water culture condition was monitored. Five days after the water culture, white root primordia of Z2 and H4 emerged from the stem epidermis section in the water, and more root primordia were observed on Z2 than that on H4. At the seventh and the tenth day, there were significantly more root primordia on Z2 than that on H4 (Figure 1).

### 3.2. Transcriptome Sequencing Analysis of Twelve cDNA Libraries

In order to study the molecular mechanism of rooting difference between Z2 and H4, mRNA-Seq analysis was performed on stems with root primordium induced by tap water (treatment) and stems without root primordium (control). Three biological replicates were set for treatment and control, respectively, with a total of 12 cDNA libraries (Z2CK1, Z2CK2, Z2CK3, Z2T1, Z2T2, Z2T3, H4CK1, H4CK2, H4CK3, H4T1, H4T2, and H4T3). The transcriptome sequencing analysis yielded 38.17–50.74 million raw reads per cDNA library with a total of 528.2 million raw read for all cDNA libraries (Table 1). After removing low quality reads and reads containing adapters or poly (N), an average of 5.8 Gb clean data were obtained for each replicate. For all 12 cDNA libraries, the percentage of phred scores at Q20 and Q30 levels exceeded 97.65% and 93.27%, respectively (Table 1), suggesting high quality of transcript data. The remaining clean reads were mapped to the *Boehmeria nivea* (L.) genome (data unpublished) using HISAT (Hierarchical Indexing for Spliced Alignment of Transcripts) software. The result showed that more than 90.23% of the clean reads were mapped onto the ramie genome for all cDNA libraries, and over 63.27% were observed to be unique mapped reads (Table 1), indicating that the samples were comparable.

### 3.3. Comparison of Gene Expression Pattern between Z2 and H4

**Figure 2:**
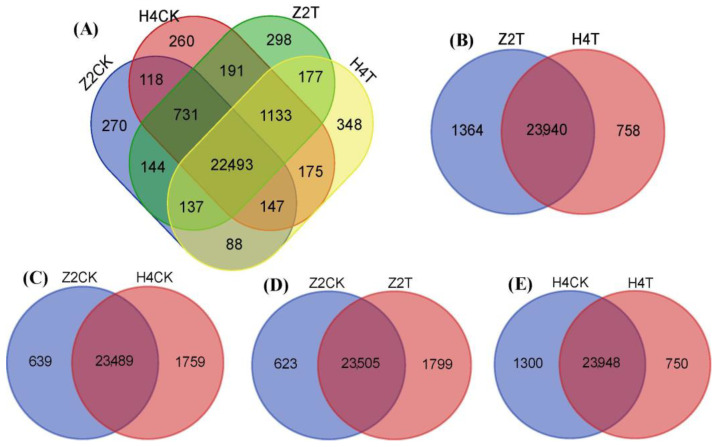
Venn diagram of all genes in the 12 cDNA libraries. H4CK, control of Huazhu No 4; H4T, treatment of Huazhu No 4; Z2CK, control of Zhongzhu No 2; Z2T, treatment of Zhongzhu No 2. (**A**) Statistics of all the genes identified in H4CK, H4T, Z2CK, and Z2T. (**B**) Comparison of gene number between Z2T and H4T. (**C**) Comparison of gene number between Z2CK and H4CK. (**D**) Comparison of gene number between Z2CK and Z2T. (**E**) Comparison of gene number between H4CK and H4T.

A total of 26,723 genes, including 25,861 known genes and 862 novel genes, were identified in the 12 cDNA libraries (Additional file 2: sheet2), with 25,248, 24,698, 24,128, and 25,304 in H4CK, H4T, Z2CK, and Z2T, respectively. Most of the genes were between 300–1600 bp in length, and the number of genes longer than 3000 bp was the highest, accounting for 7% of all genes. There were 22,493 common genes in H4CK, H4T, Z2CK, and Z2T (Figure 2A), denoting most of the genes having similar expression behavior in the four libraries. In the treatment group (H4T vs. Z2T), there were 23,940 common genes between Z2T and H4T, while there were 1364 and 758 distinctive genes in Z2T and H4T, respectively (Figure 2B). In the control group (H4CK vs. Z2CK), the number of the distinctive genes in Z2CK and H4CK were 639 and 1759, respectively (Figure 2C). For Z2, the number of distinctive genes in Z2T was about three times that of Z2CK, but in H4, the number of distinctive genes in H4T was 0.58 times that of H4CK (Figure 2D,E). These results may provide us with clues about why the two varieties have different rooting patterns.

### 3.4. Identification and Expression of DEGs

**Figure 3:**
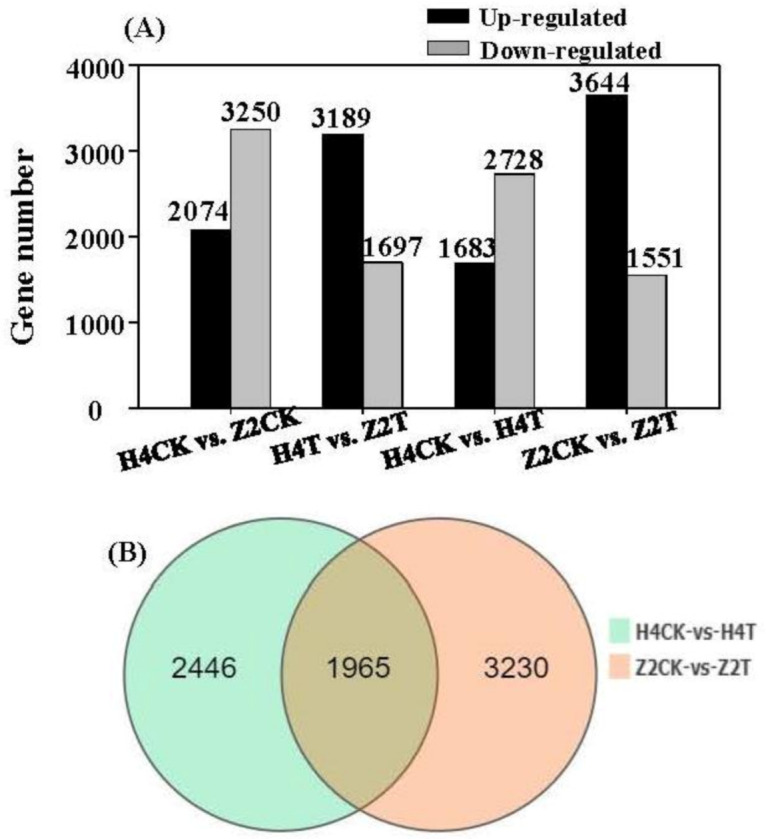
(**A**) Number of upregulated and downregulated differentially expressed genes (DEGs) in four pairwise comparisons (H4CK vs. Z2CK, H4T vs. Z2T, H4CK vs. H4T, and Z2CK vs. Z2T). (**B**) Venn diagram of DEGs in the pairwise comparisons of H4CK vs. H4T and Z2CK vs. Z2T.

To determine the genes that were differently expressed in the two genotypes, four pairwise comparisons (H4CK vs. Z2CK, H4T vs. Z2T, H4CK vs. H4T, and Z2CK vs. Z2T) were performed. DEGs were identified by the threshold of *P*_adj_ value ≤ 0.001 and fold change ≥ 2. A total of 5324, 4886, 4411, and 5195 DEGs were identified in the comparison of H4CK vs. Z2CK, H4T vs. Z2T, H4CK vs. H4T, and Z2CK vs. Z2T, respectively (Figure 3A). In the control group (H4CK vs. Z2CK), 2074 up- and 3250 downregulated DEGs were identified in Z2CK, while in the treatment group (H4T vs. Z2T), 3189 up- and 1697 downregulated DEGs were found in Z2T, suggesting different gene expression patterns in the two genotypes resulting from water treatment. In the comparisons of H4CK vs. H4T and Z2CK vs. Z2T, there were 1683 up- and 2728 downregulated DEGs in H4, while there were 3644 up- and 1551 downregulated DEGs in Z2, indicating that more genes were induced in Z2, but more genes were inhibited in H4. The comparisons of H4CK vs. H4T and Z2CK vs. Z2T shared 1965 common genes, and there were 2446 and 3230 distinctive genes expressing in the comparison of Z2CK vs. Z2T and the comparison of H4CK vs. H4T, respectively (Figure 3B).

### 3.5. GO (Gene Ontology) Clustering of DEGs

**Figure 4:**
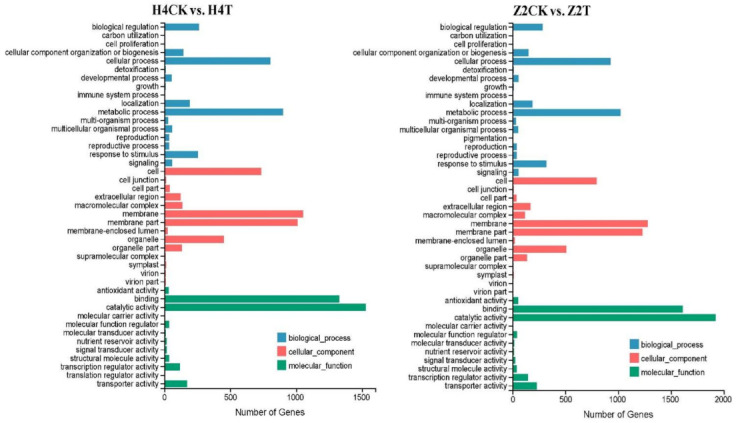
Gene ontology (GO) classification of DEGs in the pairwise comparisons of H4CK vs. H4T and Z2CK vs. Z2T.

**Figure 5:**
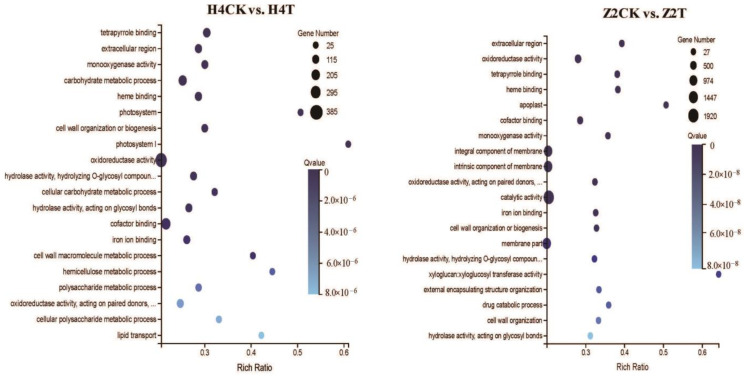
Gene ontology (GO) enrichment of DEGs in the pairwise comparisons of H4CK vs. H4T and Z2CK vs. Z2T.

**Figure 6:**
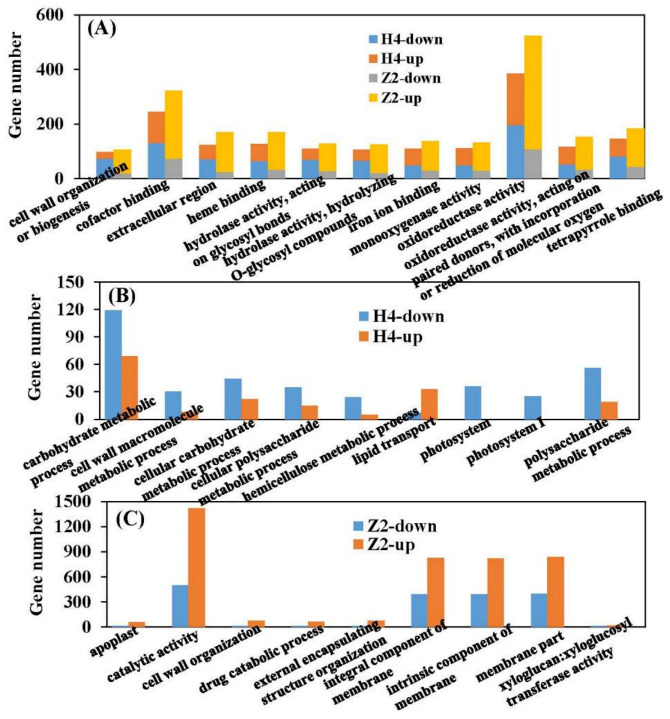
Statistical DEG number of each GO-enriched term in the pairwise comparisons of H4CK vs. H4T and Z2CK vs. Z2T. (**A**) The up- and downregulated DEG number of each common GO term between the comparisons of H4CK vs. H4T and Z2CK vs. Z2T. (**B**) The up- and downregulated DEG number of the distinctive GO terms in the comparison of H4CK vs. H4T. (**C**) The up- and downregulated DEG number of the distinctive GO terms in the comparison of Z2CK vs. Z2T. H4-up and H4-down denote up- and downregulated DEGs in the comparison of H4CK vs. H4T, respectively. Z2-up and Z2-down denote up- and downregulated DEGs in the comparison of Z2CK vs. Z2T, respectively.

GO assignment was used to classify the functions of DEGs, and significantly enriched GO term was defined when *P*_adj_ value was less than 0.05. In total, 2879 DEGs (1146 up- and 1733 downregulated) of the H4 group (H4CK vs. H4T) were assigned into 43 enriched GO terms consisting of 17 biological processes (metabolic process, cellular process, biological regulation, etc.), 14 cellular components (membrane, membrane part, cell, etc.), and 12 molecular functions (catalytic activity, binding, translation regulator activity, etc.) (Figure 4). Meanwhile, 3485 DEGs (2500 up- and 985 downregulated) in the Z2 group (Z2CK vs. Z2T) were assigned into 43 enriched GO terms consisting of 18 biological processes (metabolic process, cellular process, pigmentation, etc.), 14 cellular components (membrane, membrane part, cell, etc.), and 11 molecular functions (catalytic activity, binding, transporter activity, etc.) (Figure 4). Eighty-nine and 106 significantly enriched GO terms were identified in the H4 group and the Z2 group, respectively (Additional file 3: sheet3). The top 20 significantly enriched GO terms sorted by *P*_adj_ value from small to large are shown in Figure 5. There were 11 common significantly enriched GO terms between the H4 group and the Z2 group, such as cell wall organization or biogenesis, oxidoreductase activity, hydrolase activity acting on glycosyl bonds, cofactor binding, and others. In each common GO term, the total number of DEGs in the Z2 group was higher than that in the H4 group, and the number of upregulated DEGs was higher than the downregulated DEGs in the Z2 group but lower in the H4 group (Figure 6A). Nine distinctive significantly enriched GO terms were found in the H4 group, which mainly included the polysaccharide metabolic process, carbohydrate metabolic process, cellular carbohydrate metabolic process, cell wall macromolecule metabolic process, photosystem, and others (Figure 6B). The expression of the most DEGs in these distinctive GO terms was inhibited, suggesting that the photosynthesis process and the cell wall formation process may be suppressed in the process of root primordium formation in H4. Meanwhile, other nine significantly enriched GO terms were found in the Z2 group, mainly including cell wall organization, the integral component of membrane, the membrane part, external encapsulating structure organization, and others (Figure 6C). The number of upregulated DEGs in these distinctive GO terms was more than that of downregulated DEGs, indicating that cell wall formation may promote root primordium formation in Z2.

### 3.6. KEGG Pathway Analysis of DEGs

**Figure 7:**
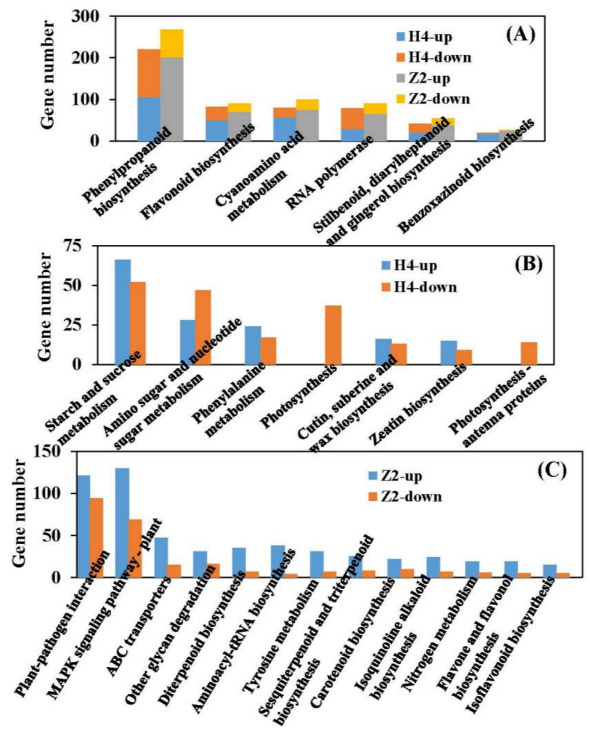
Statistical DEG number of each pathway identified by Kyoto Encyclopedia of Genes and Genomes (KEGG) analysis. (**A**) The up- and downregulated DEG number of each common pathway between the comparisons of H4CK vs. H4T and Z2CK vs. Z2T. (**B**) The up- and downregulated DEG number of the distinctive pathways in the comparison of H4CK vs. H4T. (**C**) The up- and downregulated DEG number of the distinctive pathways in the comparison of Z2CK vs. Z2T. H4-up and H4-down denote up- and downregulated DEGs in the comparison of H4CK vs. H4T, respectively. Z2-up and Z2-down denote up- and downregulated DEGs in the comparison of Z2CK vs. Z2T, respectively.

**Figure 8:**
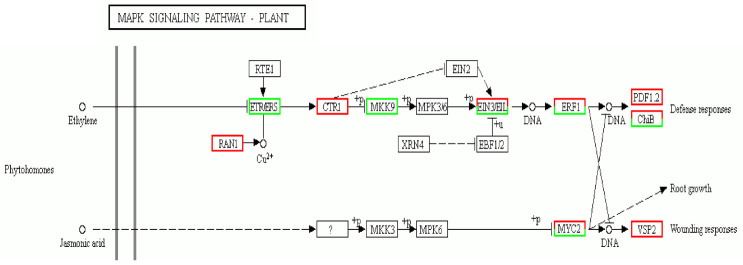
The MAPK signaling pathway identified in the comparison of Z2CK vs. Z2T. Red and green denote the up- and downregulated DEGs, respectively. RAN1, responsive to antagonist1; RTE1, reversion to ethylene sensitivity1; ETR, ethylene response; ERS, ethylene response sensor; CRT1, constitutive triple response1; MKK, MAP kinase kinase; MPK, mitogen-activated protein kinase; EIN, ethylene-insensitive; EIL, EIN3-like; EBF1/2, EIN3 binding F-box1/2; ERF1, ethylene response factor1; PDF1.2, plant defensin1.2; ChiB, basic chitinase; MYC2, jasmonate-insensitive1; VSP, vegetative storage protein; XRN4, exoribonuclease4.

To further the insight into the molecular interactions among the DEGs, KEGG analysis was performed and the significantly enriched pathway was defined when the *P*_adi_ value was less than 0.05. A total of 1966 (in H4 group: H4CK vs. H4T) and 2381 (in Z2 group: Z2CK vs. Z2T) DEGs were enriched into 133 and 131 pathways, respectively (Additional file 4: sheet4). Thirteen and 19 significant enriched pathways were identified in the H4 group and the Z2 group, respectively. Among them, there were six common pathways between the two groups, while there were seven (photosynthesis, photosynthesis antenna proteins, starch and sucrose metabolism, etc.) and 13 (MAPK signaling pathway–plant, carotenoid biosynthesis, plant–pathogen interaction, etc.) distinctive pathways in the H4 group and the Z2 group, respectively (Figure 7A). In the MAPK signaling pathway of the Z2 group, ethylene and jasmonic acid (JA) signaling pathways were identified (Figure 8).

More DEGs were induced in the Z2 group (1033 up- and 404 downregulated), while more DEGs were reduced in the H4 group (424 up- and 434 downregulated). The number of downregulated DEGs in photosynthesis, photosynthesis antenna proteins, and amino sugar and nucleotide sugar metabolism pathways in the H4 group were more than that of upregulated DEGs (Figure 7B), suggesting that the photosynthesis process may be inhibited in H4 during root primordium formation. In each distinct pathway of the Z2 group, the number of upregulated DEGs was more than that of downregulated, especially in MAPK signaling pathway–plant, the number of upregulated DEGs was about two times that of downregulated DEGs (Figure 7C).

### 3.7. Verification of Expression of DEGs by qPCR

**Figure 9:**
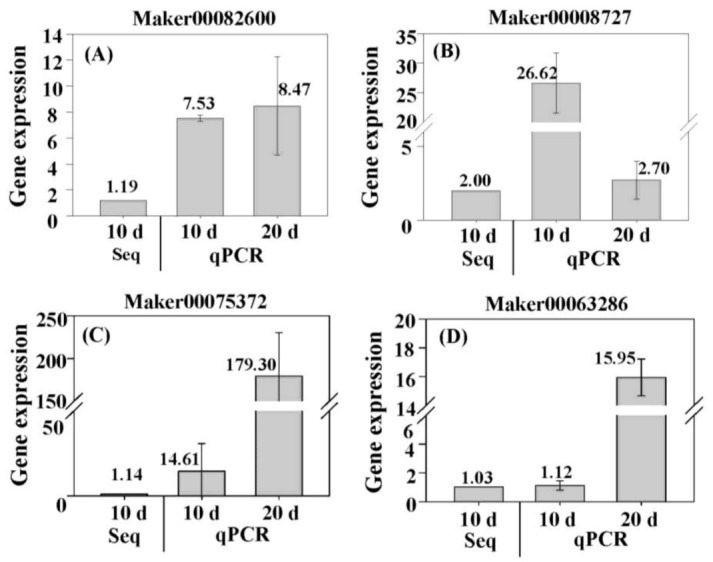
qPCR analysis of randomly selected DEGs in stems of ramie under the tap water-cultured condition. (**A**) Expression of Maker00082600 in Zhongzhu No 2 cultured with tap water (Z2T). The 2^−ΔΔCt^ method was used to calculate the relative expression. The expression in Z2CK (without tap water culture) was used as the control, and its value was set to 1. (**B**) Expression of Maker00008727 in Huazhu No 4 cultured with tap water (H4T). The expression in H4CK (without tap water culture) was used as the control, and its value was set to 1. (**C**) and (**D**) expression of Maker00075372 and Maker00063286 in Z2T. The expression in H4T was used as the control, and its value was set to 1. For (**A**) to (**D**), data are means ± SD of three replicates. “Seq” denotes the expressions obtained by mRNA-Seq analysis. “qPCR” denotes the expressions obtained by qPCR.

Four DEGs (Maker00082600, Maker00008727, Maker00075372, and Maker00063286) were randomly selected for qPCR to test the reliability of expression of DEGs in sequencing results. The results showed that there was good agreement between the mRNA-Seq data and the qPCR data (Figure 9). After 20 days of hydroponics, the expression of Maker00082600, Maker00075372, and Maker00063286 was significantly higher than that of 10 days of hydroponics, while the expression of Maker00008727 was lower than that of 10 days of hydroponics, suggesting different expression patterns of different DEGs during root primordium formation in ramie.

## 4. Discussion

Root generation is the key factor for growth and development of asexual propagation plants [25]. Studying the molecular mechanism of rooting of ramie cuttings is helpful to improving the survival rate of ramie cuttings. However, little is known about the molecular mechanism of rooting in ramie cuttings. In this study, mRNA-Seq analysis was performed to reveal the regulatory mechanism of AR in ramie cuttings using two ramie varieties (H4 and Z2) with different rooting patterns before rooting (control, CK) and 10 days water-induced adventitious rooting (treatment, T). Gene expression patterns and expression of DEGs between the two varieties and within the two varieties are discussed.

In total, the expression of 26,723 genes was detected in all the sequenced samples, and most of the genes (22,493) were commonly expressed in H4CK, H4T, Z2CK, and Z2T (Figure 2A). More distinctive genes were expressed in Z2T (1799) than that in Z2CK (623), while fewer distinctive genes were observed in H4T (750) compared to H4CK (1300) (Figure 2D,E), denoting different gene expression patterns in the two varieties after tap water culture. There were more distinctive genes expressed in H4CK than in Z2CK, but differently, there were fewer distinctive genes in H4T than in Z2T (Figure 2B,C). A total of 5324, 4886, 4411, and 5195 DEGs were identified in the comparison of H4CK vs. Z2CK, H4T vs. Z2T, H4CK vs. H4T, and Z2CK vs. Z2T, respectively (Figure 3A). After tap water culture, more DEGs were induced in Z2 (3644 up- and 1551 down-regulated DEGs in Z2CK vs. Z2T) but more DEGs were inhibited in H4 (1683 up- and 2728 down-regulated DEGs in H4CK vs. H4T). These different expression patterns of all genes and DEGs between H4 and Z2 provide clues to the regulatory mechanism of their different rooting patterns.

There were 11 common significantly enriched GO terms between the H4 group (H4CK vs. H4T) and the Z2 group (Z2CK vs. Z2T), including cell wall organization or biogenesis, oxidoreductase activity, hydrolase activity acting on glycosyl bonds, cofactor binding, and others. Interestingly, in each common significantly enriched GO term, more DEGs were induced in the Z2 group (2500 up- and 985 downregulated), but more DEGs were inhibited in the H4 group (1146 up- and 1733 downregulated) (Figure 6A), indicating the probable reasons of the different rooting patterns in the two varieties. Cell wall modification is an important biological process of root formation [26]. In the H4 group, 30 DEGs related to the cell wall macromolecule metabolic process were upregulated and 8 DEGs were downregulated, while in the Z2 group, 73 and 15 DEGs related to cell wall organization were upregulated and downregulated, respectively (Figure 6B). These results suggested different molecular mechanisms of cell wall formation in the two varieties, which may result in different rooting patterns.

Photosynthesis plays an important role in root occurrence and root growth [27,28]. In *Pinus taeda*, genes related to photosynthesis are downregulated during the root initiation phase [29]. In this study, two distinctive significantly enriched GO terms—photosystem and photosystemⅠ—were identified in the H4 group, and genes involved in these GO terms were downregulated, which was consistent with the previous study. In addition, KEGG analysis showed that two significant pathways (photosynthesis and photosynthesis antenna proteins) were distinctively identified in the H4 group (Figure 7B), and all DEGs of the two pathways were downregulated. Most of the raw materials in root formation and development, such as sugars, are produced by photosynthesis. According to the downregulated DEGs related to photosynthesis, we speculated that H4 may show weak photosynthetic capacity, which may result in fewer root primordia.

Phytohormones play an important role in regulating rooting of cuttings [10,14,30]. Auxin is the primary plant hormone to promote AR in plant, and it has been found to positively regulate the occurrence and number of AR in many species [31,32,33]. Ethylene, salicylic acid, and brassinolide can also positively promote the occurrence and number of AR [12,34,35], while strigolactones, abscisic acid, gibberellins, and cytokinins negatively regulate the occurrence and number of AR [36,37,38,39]. JA has been found to synergize with auxin to promote the occurrence of AR formation in potato and *Petunia* [40,41], but it has also been found to inhibit the occurrence of AR in *Arabidopsis* [42]. In this study, the MAPK signaling pathway–plant pathway was distinctively significantly identified in the Z2 group, which showed that JA can interact with ethylene to regulate the occurrence and number of AR in ramie cuttings (Figure 8). Ethylene and JA are two regulators that modulate local responses to wounding in *Nicotiana attenuata* [43]. It has been described that during AR emergence, which means breakage of tissue, plants produce compounds with antimicrobial activities to avoid infection by bacteria. The interaction between JA and ethylene in ramie cuttings may modulate the breakage of tissue, which may be one of the reasons for more AR formation in Z2.

## 5. Conclusions

Here, we provide a global view of the transcriptomic divergence of two ramie varieties with different rooting patterns before rooting and 10 days water-induced adventitious rooting using mRNA-Seq. Cell wall formation, the photosynthesis process, and the JA signaling pathway were identified to regulate AR formation in ramie cuttings. Our results provide new insight into mechanisms of AR formation in different ramie varieties.

## Figures and Tables

**Table 1:** Statistical analysis of transcriptome data in the 12 cDNA libraries. H4, Huazhu No 4; Z2, Zhongzhu No 2; T1, T2 and T3 denote the three biological replicates of the treatment (cultured with tap water); CK1, CK2, and CK3 denote the three biological replicates of the control (without water treatment).

Sample	Total Raw Read (M)	Total Clean Read (M)	Total Clean Base (Gb)	Clean Read q20 (%)	Clean Read q30 (%)	Total Mapping Genome Ratio (%)	Uniquely Mapping Genome Ratio (%)
H4T1	41.10	36.03	5.44	97.78	93.58	90.51	63.70
H4T2	47.88	42.30	6.39	97.84	93.73	90.35	63.64
H4T3	39.33	34.14	5.15	97.70	93.39	90.23	63.27
H4CK1	45.76	40.17	6.06	97.81	93.70	90.37	65.14
H4CK2	50.74	44.39	6.70	97.78	93.59	90.80	63.98
H4CK3	45.36	39.87	6.02	97.80	93.62	90.67	64.03
Z2T1	47.07	40.79	6.16	97.69	93.37	90.92	63.61
Z2T2	44.86	39.44	5.96	97.81	93.64	90.47	65.14
Z2T3	44.13	38.43	5.80	97.73	93.47	91.33	65.72
Z2CK1	38.17	33.09	5.00	97.67	93.31	90.87	65.43
Z2CK2	43.06	37.19	5.62	97.65	93.27	91.00	65.50
Z2CK3	40.74	35.38	5.34	97.70	93.41	91.43	66.06
Mean	44.02	38.44	5.80	97.75	93.51	90.75	64.60
Total	528.20	461.22	69.64				

## Data Availability

All data presented in this study are provided either in the manuscript or additional files. Illumina sequencing data was deposited in the NCBI GEO database with accession number GSE116063.

## Supplementary Materials

The following are available online at https://www.mdpi.com/2223-7747/10/1/160/s1, Additional file 1 sheet1. Primer sequences of target genes selected for qPCR. Additional file 2 sheet2. Summary of the gene number in the 12 cDNA libraries. Additional file 3 sheet3. Significantly enriched gene ontology (GO) terms in the pairwise comparisons of H4CK vs. H4T and Z2CK vs. Z2T. Additional file 4 sheet4. Pathways in the comparisons of H4CK vs. H4T and Z2CK vs. Z2T identified by Kyoto Encyclopedia of Genes and Genomes (KEGG) analysis. Green parts denote significantly at *P*_adj_-value < 0.05.

## Abbreviations

AR	adventitious root
DEGs	differentially expressed genes
GO	gene ontology
JA	jasmonic acid
KEGG	Kyoto Encyclopedia of Genes and Genomes
qPCR	quantitative PCR.
